# Systemic Immune Dysregulation in Early Breast Cancer Is Associated With Decreased Plasma Levels of Both Soluble Co-Inhibitory and Co-Stimulatory Immune Checkpoint Molecules

**DOI:** 10.3389/fimmu.2022.823842

**Published:** 2022-05-23

**Authors:** Bernardo L. Rapoport, Helen C. Steel, Nomsa Hlatshwayo, Annette J. Theron, Pieter W. A. Meyer, Simon Nayler, Carol-Ann Benn, Teresa Smit, Luyanda L. I. Kwofie, Liezl Heyman, Ronald Anderson

**Affiliations:** ^1^Department of Immunology, Faculty of Health Sciences, University of Pretoria, Pretoria, South Africa; ^2^Medical Oncology Centre of Rosebank, Johannesburg, South Africa; ^3^Department of Immunology, Tshwane Academic Division, National Health Laboratory Service, Pretoria, South Africa; ^4^Drs Gritzman & Thatcher Inc. Laboratories, Johannesburg, South Africa; ^5^University of the Witwatersrand Donald Gordon Medical Centre, Johannesburg, South Africa; ^6^Netcare Breast Care Centre, Johannesburg, South Africa

**Keywords:** breast cancer, CTLA-4, co-inhibitory and co-stimulatory immune checkpoints, immune dysregulation, CD28, GITR, PD-1/PD-L1, TIM-3

## Abstract

Breast cancer cells exploit the up-regulation or down-regulation of immune checkpoint proteins to evade anti-tumor immune responses. To explore the possible involvement of this mechanism in promoting systemic immunosuppression, the pre-treatment levels of soluble co-inhibitory and co-stimulatory immune checkpoint molecules, as well as those of cytokines, chemokines, and growth factors were measured in 98 newly diagnosed breast cancer patients and compared with those of 45 healthy controls using multiplex bead array and ELISA technologies. Plasma concentrations of the co-stimulatory immune checkpoints, GITR, GITRL, CD27, CD28, CD40, CD80, CD86 and ICOS, as well as the co-inhibitory molecules, PD-L1, CTLA-4 and TIM-3, were all significantly lower in early breast cancer patients compared to healthy controls, as were those of HVEM and sTLR-2, whereas the plasma concentrations of CX3CL1 (fractalkine), CCL5 (RANTES) and those of the growth factors, M-CSF, FGF-21 and GDF-15 were significantly increased. However, when analyzed according to the patients’ breast cancer characteristics, these being triple negative breast cancer (TNBC) vs. non-TNBC, tumor size, stage, nodal status and age, no significant differences were detected between the plasma levels of the various immune checkpoint molecules, cytokines, chemokines and growth factors. Additionally, none of these biomarkers correlated with pathological complete response. This study has identified low plasma levels of soluble co-stimulatory and co-inhibitory immune checkpoint molecules in newly diagnosed, non-metastatic breast cancer patients compared to healthy controls, which is a novel finding seemingly consistent with a state of systemic immune dysregulation. Plausible mechanisms include an association with elevated levels of M-CSF and CCL5, implicating the involvement of immune suppressor cells of the M2-macrophage/monocyte phenotype as possible drivers of this state of systemic immune quiescence/dysregulation.

## Introduction

Advanced solid malignancies are associated with generalized systemic immunosuppression (1) that may predispose not only for tumor progression and spread, but also for the development of serious microbial and viral infections (2–4). Notably, however, the existence of cancer-related systemic immunosuppression, seemingly from a much earlier stage in the process of tumorigenesis than was previously believed, has recently been convincingly demonstrated both in murine models of experimental tumorigenesis (5, 6) and in a number of clinical studies, mostly focused on dendritic cell (DC) dysfunction in various types of cancer (1, 7–13). In the former context, the preclinical study reported by Allen et al. (6) is particularly noteworthy. These researchers investigated alterations in the cellularity of paired tumor tissue and blood following experimental induction of various types of tumors spanning five different anatomical sites including breast cancer, melanoma, colorectal cancer, glioma, and pancreas cancer in mice. The tumor-bearing animals manifested systemic immunosuppression that was evident irrespective of tumor type and most consistent in the setting of breast cancer. Systemic immunosuppression was characterized by elevated levels of the pro-inflammatory cytokines, interleukin (IL)-1α and granulocyte colony-stimulating factor (G-CSF), as well as abnormalities of systemic DC phenotype and function (6). This resulted in impairment of cytotoxic CD8^+^ T cell function and an adverse outcome following experimental infection with the bacterial and viral pathogens, *Listeria monocytogenes*, and lymphocytic choriomeningitis virus, respectively. Importantly, this systemic inflammatory phenotype and associated immunosuppression were reversed by tumor resection (6).

The findings of Allen et al. (6) are in keeping with gene expression microarray data sourced from the earlier “Norwegian Women and Cancer (NOWAC)” study (14). By profiling 16,792 “unique” genes expressed in matched blood and tumor tissue from 173 breast cancer patients, the authors of the study identified a strong association between the systemic and intra-tumoral immune responses driven by the primary tumor (14).

Given that next generation immunotherapies target the most intransigent types of malignancy, overcoming the apparent additional obstacle of systemic immunosuppression represents a daunting challenge, underscoring the necessity of implementation of extensive systemic immune/inflammatory protein profiling of cancer patients. Such a strategy may enable the identification of the most prominent mediators of tumor-driven systemic immunosuppression/immune dysregulation, as well as the types of cancer and stages of the disease in which they are operative. In the current seemingly novel study, we have focused on previously untreated, early breast cancer, primarily targeting a broad spectrum of soluble co-inhibitory and co-stimulatory immune checkpoint molecules (n=16) and their relationships with anti-inflammatory and pro-inflammatory cytokines (n=10), as well as chemokines (n=7) and growth factors (n=6). Our data with respect to alterations in the levels of soluble immune checkpoints are seemingly consistent with the existence of significant, systemic immune dysregulation, even in early-stage breast cancer.

## Patients and Control Subjects

Female patients (n=98, mean age ± SD = 52.46 ± 12.80 years; range = 27-84 years) with early breast cancer attending the Medical Oncology Centre of Rosebank, Johannesburg, South Africa, who were deemed eligible for neo-adjuvant therapy, were recruited to the study. Eligibility criteria included: i) age ≥18 years; ii) Eastern Cooperative Oncology Group (ECOG) performance status (PS of 0, 1 or 2) (15); iii) histologically confirmed breast cancer (16) classified as clinical stage I, II or III according to the AJCC Breast Staging 8^th^ Edition (17); iv) normal bone marrow, liver and renal function; v) neo-adjuvant chemotherapy with anthracycline (A)- and/or taxane (T)-based chemotherapy regimens; or platinum-based chemotherapy added to AT based therapies. Human epidermal growth factor receptor 2 (Her2) -positive patients received neo-adjuvant trastuzumab-based treatment.

Exclusion criteria were as follows: i) prior systemic chemotherapy for breast cancer within 5 years; ii) a history of any other malignancy during the preceding five years, with the exception of basal cell or squamous cell carcinoma of the skin treated with local resection only, as well as carcinoma *in situ* of the cervix; iii) patients with confirmed stage IV disease; iv) known seropositivity for human immunodeficiency virus (HIV) and/or hepatitis B or hepatitis C viruses; v) uncontrolled intercurrent illness including, but not limited to, active infection, symptomatic congestive heart failure, unstable angina pectoris, cardiac arrhythmia or psychiatric illness/social situations that would limit compliance with study requirements; vi) pregnancy or breast feeding; and vii) otherwise not deemed to be a good study candidate according to the sole discretion of the principal investigator (BLR).

The group of healthy female control participants (n=45, mean age ± SD = 49.9 ± 12.08 years; range 24–70 years) was recruited almost exclusively from the female personnel of the Medical Oncology Centre of Rosebank and the Faculty of Health Sciences, University of Pretoria. Exclusion criteria included; i) those with uncontrolled medical conditions, and ii) any potential participant deemed unwell by the qualified nursing sister in attendance on the day of venepuncture.

## Ethics Committee Clearance

Permission to undertake this study and to draw blood from patients with early breast cancer and from matched, healthy control subjects was granted by the Research Ethics Committee of the Faculty of Health Sciences, University of Pretoria in full compliance with the World Medical Association Declaration of Helsinki 2013. Two submissions were approved; firstly, the breast cancer study and secondly, a submission in respect of the healthy control subjects (respective Approval Numbers 517/2017 and 762/2020). Prior, written informed consent was obtained from all participants.

## Methods

Whole blood samples of 20 mL and 10 mL volumes were collected in ethylenediaminetetraacetic acid (EDTA)-containing vacutainers from the breast cancer patients and control subjects, respectively, and the plasma promptly separated, aliquoted and stored at -80°C.

Biomarker measurements were focused primarily on the plasma concentrations of soluble co-inhibitory and co-stimulatory immune checkpoints. Other measured systemic immune biomarkers relevant to the pathogenesis of breast cancer included an array of anti-inflammatory and pro-inflammatory cytokines, chemokines, growth factors and C-reactive protein (CRP). All results are expressed as picograms (pg)/mL plasma with the exception of CRP the results of which are expressed as micrograms (µg)/mL plasma.

### Soluble Immune Checkpoints

The co-stimulatory immune checkpoint molecules assayed included: CD27; CD28; CD40; CD80; CD86; Glucocorticoid TNFR family-related receptor (GITR); GITR ligand (GITRL); and inducible T cell co-stimulator (ICOS; CD278). Co-inhibitory immune checkpoints included: B and T lymphocyte attenuator (BTLA; CD272); cytotoxic T lymphocyte-associated protein 4 (CTLA-4; CD152); programmed cell death protein 1 (PD-1; CD279); PD-1 ligand (PD-L1; CD274); lymphocyte-activation gene 3 (LAG-3; CD223); and T-cell immunoglobulin and mucin containing domain 3 (TIM-3; CD366). In addition, we measured the levels of the dual immune checkpoints, herpes virus entry mediator (HVEM; CD270) and Toll-like receptor 2 (sTLR-2; CD282).

### Cytokines

This group of immune/inflammatory mediators included the anti-inflammatory/immunosuppressive cytokines: IL-4, IL-10, IL-1 receptor antagonist (IL-1Ra), and transforming growth factor-β1 (TGF-β1). Predominantly pro-inflammatory cytokines included: IL-2, IL-6, IL-16 (augments the expression of pro-inflammatory cytokines by human monocytes), IL-17A, interferon (IFN)-α2 and IFN-γ.

Plasma levels of TGF-β1 were measured using a commercial enzyme-linked immunosorbent assay (ELISA) system (Abcam, Cambridge, MA, USA). Latent TGF-β1 was activated by the addition of 1N hydrochloric acid (HCL) to the plasma samples. After thorough mixing, the samples were incubated for 10 minutes at room temperature. The samples were then neutralized by the addition of 1.2N sodium hydroxide (NaOH) and mixed thoroughly. The samples were diluted 10-fold, and the assay was performed immediately as per the manufacturer’s instructions.

### Chemokines

The following chemokines were included: CX3CL1 (fractalkine), CXCL5 (ENA78), CXCL8 (IL-8), CXCL9 (MIG, monokine induced by gamma interferon), CXCL10 (IP-10, interferon gamma-induced protein 10), CCL23 (macrophage inflammatory protein 3), CCL26 (eotaxin-3) and CCL5 (RANTES; Regulated upon Activation, Normal T Cell Expressed and Presumably Secreted).

### Growth Factors

The six growth factors included were: macrophage colony-stimulating factor (M-CSF), granulocyte/macrophage colony-stimulating factor (GM-CSF), granulocyte colony-stimulating factor (G-CSF), vascular endothelial growth factor (VEGF), fibroblast growth factor 21 (FGF-21) and growth/differentiation factor 15 (GDF-15).

The immune checkpoint molecules, chemokines/cytokines (with the exception of TGF-β1) and growth factors were measured in the plasma samples using customized Milliplex^®^ MAP kits (Merck KgaA, Darmstadt, Germany). The growth factors (FGF-21 and GDF-15) were measured using Invitrogen® ProcartaPlex immunoassays (Thermo Fisher Scientific, Waltham, MA, USA). The remaining growth factors were measured in plasma samples (diluted 4-fold) using customized Bio-Plex^®^ immunoassay kits (Bio-Rad Laboratories Inc., Hercules, CA, USA). Analysis of these biomarkers was performed according to the manufacturer’s instructions. The biomarkers were assayed on a Bio-Rad Luminex^®^ 200™ Suspension Array System (Bio-Rad Laboratories, Inc.). Bio-Plex^®^ Manager Software 6.0 was used for bead acquisition and analysis of median fluorescence intensity.

### C-Reactive Protein

Plasma CRP levels were assayed using high-sensitivity laser nephelometry (Siemens Healthcare Diagnostics, BN Prospec Nephelometer, Newark, NJ, USA). Notably, elevated levels of CRP are associated with an increased risk of breast cancer (18).

### Expression and Statistical Analysis of Results

Plasma concentrations of the various test biomarkers are expressed as the median values with interquartile ranges (25% – 75%) expressed as 95% lower and upper confidence levels (LCL and UCL), respectively in the tables.

The primary hypothesis was that a significant difference in the plasma levels of soluble immune checkpoints, cytokines, chemokines, and growth factors existed between early breast cancer patients and healthy controls. Descriptive statistics were used to tabulate patient characteristics. The Mann-Whitney U-test was used to compare the various test biomarkers levels between breast cancer patients and healthy controls. Fisher exact or Chi-squared tests were used for the analysis of categorical variables. Spearman correlation analysis was performed to evaluate the correlations between soluble co-inhibitory and co-stimulatory immune checkpoint molecules, growth factors, chemokines and cytokines. Number Cruncher Statistical Software (NCSS) version 11 for Windows (USA) was used for statistical analyses.

## Results

Patient characteristics are shown in **Table 1**.

**Table 1:** Patient Characteristics.

**Age (n=98)**	
Median Age	52
Range	27-85
**Menopausal Status**	
Peri-menopausal	2 (2%)
Pre-menopausal	55 (56%)
Post-menopausal	41 (42%)
**Biological Type**	
Her2-positive	16 (16%)
Luminal A	2 (2%)
Luminal B	14 (14%)
TNBC	66 (68%)
**Grade**	
1	1 (1%)
2	25 (26%)
3	69 (70%)
**Stage**	
IA	13 (13%)
IIA	40 (41%)
IIIA	7 (7%)
IIB	31 (32%)
IIIB	4 (4%)
IIIC	3 (4%)
**Glands**	
Negative	67 (68%)
Positive	31 (32%)
**Estrogen Receptor Status**	
Negative	68 (68%)
Positive	31 (32%)
**Progesterone Receptor Status**	
Negative	81 (82%)
Positive	17 (17%)
**Her2 Status**	
Negative	82 (84%)
Positive	16 (16%)
**Ki-67 mean = 50% [6-100%]**	
≤14%	7 (7%)
15-39%	31 (32%)
≥40%	58 (59%)
Not done	2 (2%)

Comparisons of plasma levels of the soluble immune checkpoint proteins between early breast cancer patients and healthy controls are shown in **Table 2**, while those of the cytokines, chemokines and growth factors are shown in **Table 3**.

**Table 2:** Comparison of plasma concentrations of soluble immune checkpoints between newly diagnosed breast cancer patients and healthy control subjects.

	Soluble Immune Checkpoint Molecule	Breast Cancer (n=98)			Controls (n=45)			*p* value
		Median pg/mL	95% LCL of Median	95% UCL of Median	Median pg/mL	95% LCL of Median	95% UCL of Median	
**Co-stimulatory**	CD27	3131,29	2639,21	3568,54	4577,35	3391,13	5784,85	0,000
	CD28	32176,41	27889,65	40279,32	46135,18	27210,29	67544,10	0,002
	CD40	1464,69	1262,67	1620,90	1977,68	1404,82	2569,56	0,001
	ICOS	14364,95	11122,68	15964,40	26506,65	15897,52	31725,99	0,000
	GITR	1140,80	698,30	1660,12	3797,68	1993,96	5396,86	0,000
	GITRL	5529,80	4868,15	6407,60	7151,12	5528,36	9878,41	0,002
	CD86	11199,42	9447,21	12851,98	14297,09	9391,46	20525,14	0,011
	CD80	1613,27	1317,61	1792,55	2329,77	1395,01	3042,87	0,001
**Co-inhibitory**	PD-1	11571,18	10147,12	13426,83	14917,48	7874,92	21795,02	0,120
	PD-L1	1580,69	1198,87	1978,97	3342,62	2628,64	4750,96	0,000
	CTLA-4	1585,73	1330,19	1790,69	2618,23	1578,44	3110,47	0,001
	TIM-3	3834,44	3436,22	4132,40	5046,87	4732,72	5958,87	0,001
	LAG-3	120377,50	93854,44	138811,30	150416,00	94508,53	187997,20	0,113
	BTLA	12907,97	11108,41	17084,76	18147,26	11461,86	25180,69	0,110
Dual*	sTRL-2	24059,42	20551,28	28354,07	30477,20	20928,44	50302,64	0,014
	HVEM	1866,92	1674,84	2007,57	2290,19	2079,46	2618,44	0,000

(*co-inhibitory and co-stimulatory).

**Table 3:** Comparison of plasma concentrations of cytokines, chemokines and growth factors between newly diagnosed breast cancer patients and control subjects.

	Breast Cancer (n=98)			Controls (n=45)			*p* value
Chemokines	Median pg/mL	95% LCL of Median	95% UCL of Median	Median pg/mL	95% LCL of Median	95% UCL of Median	
CXCL5 ENA 78	535,58	250,70	763,49	2246,51	1540,24	3246,49	0,000
CCL26 Eotaxin 3	4,31	4,31	8,41	6,36	3,28	8,41	0,354
CX3CL1 Fraktalkine	445,13	399,04	489,30	397,12	366,07	431,69	0,009
CCL5 RANTES	84,22	78,32	90,09	48,72	36,30	66,96	0,000
CXCL10 IP-10	485,82	426,98	607,59	543,33	498,35	638,22	0,868
CXCL9 MIG	91,31	76,65	112,33	92,92	74,64	117,50	0,399
CCL23 MIP-3	949,97	701,29	1136,60	1476,91	1267,40	1860,61	0,049

**Cytokines**	**Median pg/mL**	**95% LCL of Median**	**95% UCL of Median**	**Median** **pg/mL**	**95% LCL of Median**	**95% UCL of Median**	***p* value**
IL-2	9,01	8,19	10,37	9,81	7,29	11,50	0,329
IL-4	126,24	102,57	156,37	146,78	113,16	200,93	0,187
IL-6	10,52	8,90	11,30	10,40	7,56	13,71	0,749
IL-8	9,61	8,18	10,87	9,81	7,49	10,34	0,204
IL-16	1931,86	1569,84	2087,37	3535,39	2932,85	3813,73	0,000
Interferon α2	174,28	152,73	192,52	199,64	176,29	214,94	0,008
IL-1 Ra	418,74	346,33	466,68	503,33	448,08	625,49	0,018
Interferon γ	59,74	51,01	66,56	69,55	44,35	80,45	0,086
IL-10	42,76	36,50	50,87	47,61	34,16	59,25	0,406
IL-17A	23,05	20,19	25,90	23,92	20,84	23,38	0,961
TGF β1	20353,26	14180,32	24904,45	23785,83	16184,42	36390,72	0,986

**Growth Factors**	**Median pg/mL**	**95% LCL of Median**	**95% UCL of Median**	**Median** **pg/mL**	**95% LCL of Median**	**95% UCL of Median**	***p* value**
FGF-21	24,36	8,64	33,80	8,64	8,64	8,64	0,001
GDF-15	806,82	741,37	879,48	430,03	368,72	467,45	0,000
M-CSF	84,41	65,74	88,98	13,34	13,34	13,34	0,000
GM-CSF	13,30	13,30	13,30	13,30	13,30	13,30	1,000
G-CSF	8,75	8,75	8,75	8,75	8,75	8,75	1,000
VEGF	9,14	2,40	13,64	8,66	2,87	13,64	0,566

### Soluble Immune Checkpoint Proteins

The median plasma levels of the co-stimulatory immune checkpoint proteins were all significantly lower compared to healthy controls. These molecules included CD27 (3131.29 vs. 4577.35 pg/mL, *p*<0.0004 for controls and patients, respectively); CD28 (32176.41 vs. 46135.18 pg/mL, *p*<0.0023); CD40 (1464.69 vs. 1977.68 pg/mL, *p<*0.0005); ICOS (14364.95 vs. 26506.65 pg/mL, *p*<0.0001); GITR (1140.80 vs. 3797.68, *p*<0.0001); GITRL (5529.80 vs. 7151.12 pg/mL, *p*<0.0023); CD80 (1613.27 vs. 2329.77 pg/mL, *p*<0.001); and CD86 (11199.42 vs. 14297.09 pg/mL, *p*<0.01).

The median plasma levels of five of the co-inhibitory immune checkpoint proteins were also significantly lower compared to healthy controls. These molecules included PD-L1 (1580.69 vs. 3342.62 pg/mL, *p*<0.0001); CTLA-4 (1585.73 vs. 2618.23 pg/mL, *p*<0.001); and TIM-3 (3834.44 vs. 5046.87 pg/mL, *p*<0.001). Additionally, several co-inhibitory immune checkpoint proteins were numerically lower compared to those of healthy controls. These molecules included PD-1 (11571.18 vs. 14917.48 pg/mL); LAG-3 (120377.50 vs. 150416.00 pg/mL); and BTLA (12907.97 vs. 18147.26 pg/mL).

In the case of TLR-2 and HVEM, the median plasma levels of these two biomarkers were significantly lower in early breast cancer patients compared to those of healthy controls these being 24059.42 vs. 30477.20 pg/mL, *p*<0.0141 and 1866.92 vs. 2290.19 pg/mL, *p*<0.0001, respectively.

### Cytokines

There were no significant differences between the median plasma levels of IL-2 (9.0 vs. 9.81 pg/mL); IL-4 (126.24 vs. 146.78 pg/mL); IL-6 (10.52 vs. 10.4 pg/mL); IL-8 (9.61 vs. 9.81 pg/mL); IL-10 (42.76 vs. 47.61 pg/mL); IL-17A (23.05 vs. 23.92 pg/mL); and TGF-β1 (20353.26 vs. 23785.83 pg/mL) between early breast cancer patients compared to healthy controls. Median plasma levels of IFN-α2 (174.27 vs. 199.64 pg/mL, *p*<0.008); IL-1Ra (418.73 vs. 503.33 pg/mL, *p*<0.02) and IL-16 (1931.86 vs. 3535.39 pg/mL, *p*<0.0001) were all significantly lower in early breast cancer patients compared to healthy controls.

### Chemokines

Median plasma levels of CXCL5 (535.58 vs. 2246.51 pg/mL, *p*<0.0001) and CCL23 (949.97 vs. 1476.91 pg/mL, *p*<0.049) were significantly lower compared to healthy controls. Median plasma levels of CCL26 (4.31 vs. 6.36 pg/mL) and CXCL10 (485.82 vs. 543.33 pg/mL) were numerically lower compared to healthy controls. Plasma levels of CX3CL1 (445.13 vs. 397.12 pg/mL, *p<*0.009) and CCL5 in particular (84.22 vs. 48.72 pg/mL, *p*<0.0001) were significantly higher compared to healthy controls, while plasma levels of CXCL9 (91.31 vs. 92.92 pg/mL, *p*<0.4) were not significantly different between breast cancer patients and healthy controls.

### Growth Factors

Plasma levels of M-CSF (84.41 vs. 13.34 mL, *p*<0.0001), FGF-21 (24.36 vs. 8.64 pg/mL, *p*<0.001) and GDF-15 (806.82 vs. 430.03 pg/mL, *p*<0.0001) for breast cancer patients and controls, respectively are shown in **Table 3**. These were significantly higher in early breast cancer patients compared to healthy controls. The median plasma concentrations of GM-CSF (13.30 vs. 13.30 pg/mL), G-CSF (8.75 vs. 8.75 pg/mL) and VEGF (9.14 vs. 8.66 pg/mL) were comparable in both groups.

### C-Reactive Protein

Eight breast cancer patients had CRP values of ≥ 10 µg/mL (range 11.6 - 22.0 µg/mL) and one in the control group (16.1 µg/mL). The respective median CRP values for the breast cancer and control groups were 1.95 (0.62 - 4.58) and 1.38 (0.62-4.23) µg/mL, respectively, which were not significantly different. Total circulating leukocyte counts and neutrophil:lymphocyte ratios (NLR) of the breast cancer patients were also within the normal ranges, the respective median values being 7.31 x 10^9^/mL blood (range 3.78 – 11.68) and 1.97.

### Correlations Between the Various Immune Checkpoints

**Figure 1:**
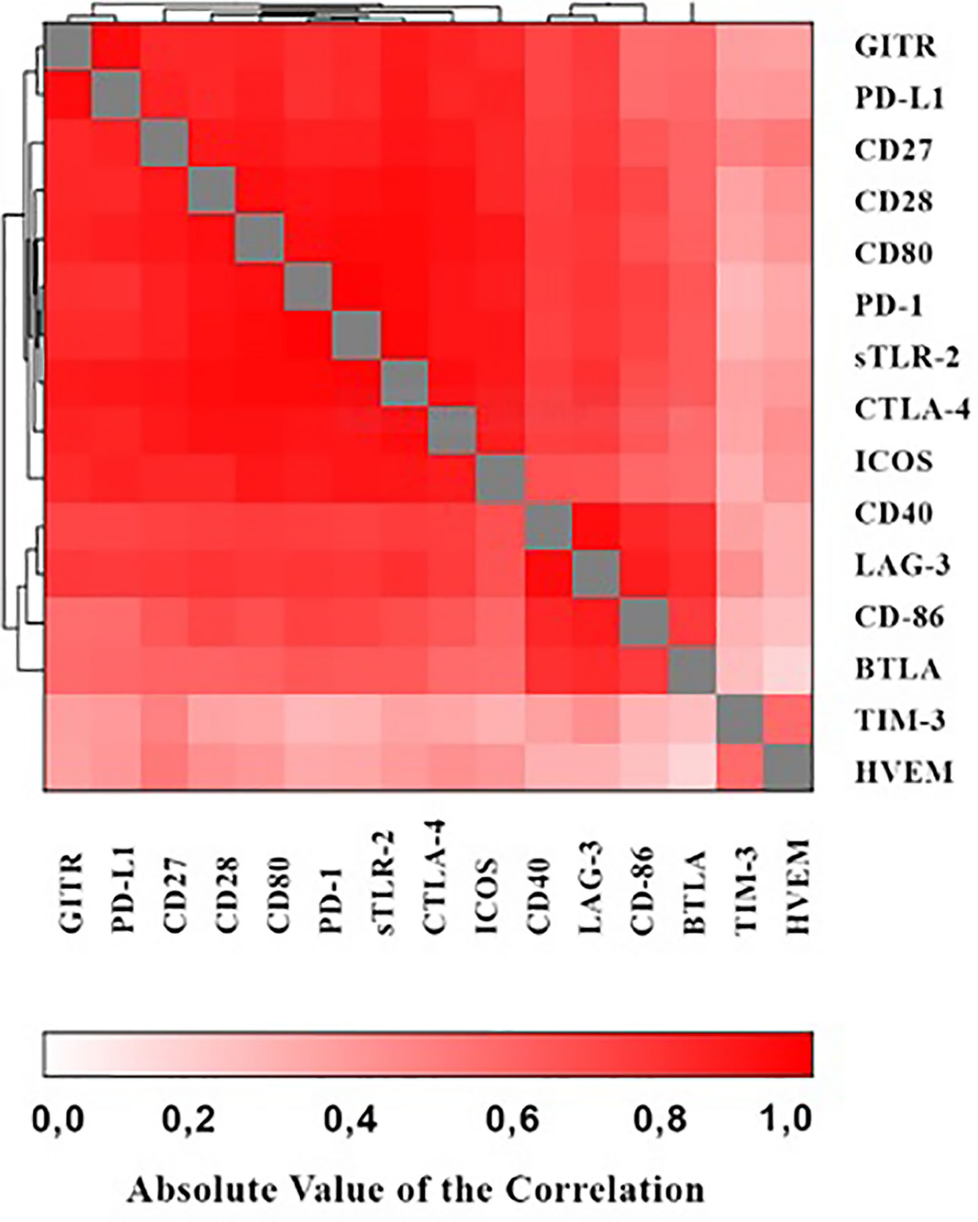
Heat map showing associations of the various immune checkpoints.

As shown in the heat map in **Figure 1** positive correlations were detected between the various immune checkpoints (with r values ranging from 0.065 - 0.983 and *p* values ranging from *p*<0.0001 – 0.476 respectively).

### Correlations of M-CSF, FGF-21, GDF-15 and CCL5 With Other Biomarkers

Correlations between the three growth factors, as well as CCL5 with i) the immune checkpoints; and ii) the cytokines/chemokines are shown in **Supplementary Tables 1** and **2**, respectively. The only correlations of note were: i) the observed significant correlations of FGF-21 with PD-1 (r = 0.32, *p*<0.008) and sTLR-2, LAG-3, GITRL, BTLA and CD86 (r = 0.25 – 0.39, *p<*0.0001 - *p<*0.026), M-CSF with GITR (r= -0.22, *p*<0.044) and PD-L1 (r = -0.24, *p*<0.030). GDF-15 showed significant correlations with TIM-3 (r = 0.18, *p*<0.055) and HVEM (r = 0.23, *p*<0.056). The significant correlations for CCL5 were with soluble immune checkpoint molecules GITR, CD27, ICOS and LAG-3 (r = 0.20 – 0.22, *p*<0.031 – *p*<0.051). Correlations with cytokines/chemokines were FGF-21 with IL-16, IFN-α2, IL-1Ra, IFN-γ, IL-6, IL-2, IL-8, IL-17A, IL-4 and CXCL5 (r = 0.32 – 0.50, *p*<0.0001 – *p*<0.042); and M-CSF with IL-16, IL-8, CXCL5 and CCL26 (r = -0.27 – 0.35, *p*<0.001 – 0.044). GDF-15 showed significant correlations with IFN-α2, IL-2 and CXCL5 (r = -0.19 – 0.14, *p*<0.013 – *p*<0.058).

### Correlations Between CCL5 and M-CSF With Platelets and Monocytes

No significant correlations between CCL5 with M-CSF (Spearman correlation: r = -0.077, p<0.7376) or of CCL5 with the circulating platelet count (r = 0.0201, *p*<0.8473) were found. The median total monocyte count was 0.53 x 10^9^/L. The median percentage of monocytes was 7.3%. Additionally, there were no correlations between monocytes and CCL5 (r = 0.06, *p*<0.563) or monocytes and M-CSF (r = 0.13, *p*<0.202).

### Correlations Between Stage and Breast Cancer Subtype With the Test Biomarkers

None of the immune checkpoint proteins, cytokines, chemokines and growth factors correlated with the stage and breast cancer subtype.

### Response to Neo-Adjuvant Chemotherapy

Patients received taxane and/or anthracycline/alkylating agent-based neo-adjuvant chemotherapy. Patients who tested positive for Her2 also received trastuzumab.

Pathological complete response (pCR) was defined as the complete disappearance of the invasive cancer in the breast and the absence of tumor in the axillary lymph nodes. There were 60 pCRs (61%) in the entire cohort. The pCR rates for Her2-positive disease, luminal disease and TNBC were 71%, 25%, and 68%, respectively.

No biomarker (soluble immune checkpoint, chemokine, cytokine, growth factor, or CRP) was predictive for pCR in this patient cohort.

## Discussion

The findings of the current study have revealed the existence of a surprisingly quiescent, systemic immune phenotype in a high proportion of patients with early, untreated breast cancer, encompassing mechanisms that drive both positive and negative regulation of cellular immune responses. In this context, it was the plasma concentrations of the group of soluble immune checkpoints that were most prominently affected in the group of breast cancer patients. Of the group of 16 soluble immune checkpoints measured, the levels of 8/8 and 3/6 co-stimulatory and co-inhibitory checkpoints, respectively, were significantly decreased. The exceptions were the inhibitory checkpoints, BTLA, LAG-3 and PD-1, the levels of which were numerically, but not significantly, lower than those of the control group.

Contrary to our expectations, we therefore failed to detect evidence of either elevated circulating levels of soluble co-inhibitory immune checkpoints, which may have driven generalized systemic immunosuppression in early breast cancer, or of a counteracting response associated with increased levels of co-stimulatory immune checkpoints. This scenario was seemingly confirmed by observations that plasma concentrations of three of the ten measured cytokines, IFN-2α, IL-16 and IL-1Ra, were significantly decreased in the group of breast cancer patients, while the concentrations of the remaining seven, including IL-10 and TGF-β1, were comparable with those of the controls. With respect to the seven measured chemokines, CXCL5 and CCL23 were substantially decreased in the group of breast cancer patients, while those of CX3CL1 (fractalkine) and CCL5, were significantly elevated. The levels of the remaining chemokines were either equivalent to, or numerically lower, than those of the controls. This apparent state of systemic immune quiescence in early breast cancer was strengthened by the observations that the median circulating leukocyte count and the NLR value were within the normal range, while the CRP levels in the groups of early breast cancer patients and control participants were also comparable.

Interestingly, the quiescent, systemic phenotype of the early breast cancer patients was associated with significantly increased plasma levels of three of the six growth factors measured. These were M-CSF, FGF-21, and GDF-15, while the levels of G-CSF, GM-CSF, and VEGF were comparable between the two groups. Aside from its key role in immune responses, M-CSF, also known as colony-stimulating factor 1, together with its receptor, M-CSFR/CSF1R, are also transiently expressed during mammary gland development, in which they play a crucial role in controlling branching morphogenesis (19, 20). Re-emergence of expression of M-CSF and its receptor in breast tissue also occurs during breast tumorigenesis, a scenario that is associated with immunosuppression and aggressive disease. Malignancies of the breast that fall into this category include primary breast cancer with nodal involvement (21–24), post-menopausal breast cancer (25), and triple-negative breast cancer (26).

Mechanisms by which M-CSF contributes to the progression of breast cancer are at least two-fold. Firstly, autocrine/paracrine activation of tumor cell proliferation *via* the M-CSF/CSF1R axis (23, 27). Secondly, by promoting recruitment of monocytes/macrophages to the breast cancer tumor microenvironment (TME). In this context, other investigators have shown that this may be achieved *via* the coordinated action of tumor-derived M-CSF and monocyte/macrophage-recruiting chemokines, such as CCL5 (28, 29), seemingly in keeping with the findings of the current study. The current study did not, however, show a correlation between plasma concentrations of CCL5 and M-CSF, suggesting that both mechanisms work independently. Under physiological conditions, platelets represent a significant source of CCL5. In our study, however, no significant correlations between circulating platelet counts and the levels of CCL5 were evident. This finding suggests that CCL5 was most likely produced by the tumor and/or other cellular elements of the TME. In the TME, tumor-associated macrophages undergo reprogramming to the M2-like, immunosuppressive, pro-metastatic phenotype *via* exposure to a range of factors such as hypoxia and lactate, as well as the cytokines, IL-10, IL-35 and TGF-β1 (30–33). However, in the current study, neither TGF-β1 nor IL-10 appeared to be operative in maintaining systemic immune quiescence. In this context, it is noteworthy that M-CSF, utilizing various mechanisms, has been reported in several studies to promote M2 macrophage polarization (34–36).

Like M-CSF, both FGF-21 and GDF-15, as described in the current study, have been reported to be significantly elevated in the blood of female breast cancer patients, albeit based on very limited prior data. In one study, Knott et al. reported significantly elevated levels of serum FGF-21 in a cohort of breast cancer patients [increased levels noted in 37/45 (82,2%) patients relative to those of a group of healthy control subjects (n=51); the respective median serum FGF-21 concentrations of the two groups were 224.56 and 76.86 pg/mL (*p*<0.0001)] (37). As with M-CSF, breast cancer cells also produce FGFs, including FGF-21, as well as expressing the FGFR1, thereby enabling autocrine tumor cell proliferation (38, 39). Activation of FGFR1 on breast cancer cells, as well as on stromal cells in the TME, initiates the production of the chemokine CX3CL1 (fractalkine) that recruits CX3CR1-expressing macrophages/monocytes to the TME (38). Here, these cells are exposed to FGF-21, as well as an array of other mediators, including various other FGFs, that promote macrophage polarization to the M2-type phenotype (40, 41). In this context, it is noteworthy that plasma levels of CX3CL1 were moderately, albeit significantly, increased in our patient cohort.

Likewise, few studies have focused on the levels of systemic GDF-15 in breast cancer patients. In one of these, encompassing various types of malignancy, including a small number of breast cancer patients (n=10), elevated serum levels of GDF-15 were detected in 6/10 patients, with the median value being significantly higher than that of a much larger group of control subjects (n=260) (42). In another similar study that also focused on various types of malignancy, including 146 breast cancer patients, but without a control group, serum GDF-15 was identified as a biomarker that was significantly associated with all-cause mortality [adjusted hazard ratio (HR)=5.47 (95% CI:2.66–11.24; *p*<0.001] (43).

Importantly breast cancer cells, as well as M2-like macrophages and fibroblasts in the TME, also produce GDF-15, which, in turn, interacts with the recently described orphan receptor GFRAL (GDNF family receptor alpha-like) expressed on tumor tissue to promote autocrine and paracrine tumor cell proliferation (44–46). Growth/differentiation factor-15 is not only produced by M2-like macrophages, but also drives the transition of these cells to this phenotype, intensifying immune suppression, fibrosis and immune exclusion in the TME (47, 48).

Prior studies have demonstrated an expansion of the CD163-expressing, M2-like macrophage population in the TME of breast cancer patients, with a high intra-tumoral density of these cells being associated with decreased survival (49). Moreover, in the context of the current study, intra-tumoral dominance of macrophages of the M2 phenotype has also been reported to impact on the phenotype of blood monocytes of breast cancer patients (49, 50). In this setting the numbers of these circulating M2-like monocytes are not only significantly increased relative to those of healthy control subjects and patients with benign disease (49, 50), but are also metabolically quiescent, displaying transcriptomes on activation that reflect decreased expression of gene sets involved in oxidative phosphorylation, fatty acid metabolism, and inner mitochondrial membrane protein complex (49). A similar association between an intra-tumoral and systemic predominance of M2-like macrophages/monocytes associated with a poor prognosis has also been described in glioma/glioblastoma patients (51, 52).

Irrespective of the potential mechanisms that drive systemic immune dysregulation, the findings of the current study may have translational implications for the efficacy, or lack thereof, of co-inhibitory immune checkpoint-targeted therapy in early breast cancer. In this context, it is noteworthy that a very recent study to which patients (n = 1174) with early triple negative breast cancer were recruited described significantly improved event free survival of up to 39% following addition of pembrolizumab to neo-adjuvant chemotherapy (53). On the other hand, however, the authors of a systematic review and meta-analysis encompassing 27 studies and 1746 patients concluded that metastatic breast cancer responded only modestly to co-inhibitory immune checkpoint-targeted therapy (54). Based on the current study, countering these variable responses of breast cancer to this immunotherapeutic strategy may necessitate overcoming the apparent failure of upregulation of co-stimulatory molecules that promote activation of anti-tumor cytotoxic T cells. On the basis of previous studies, it seems that this type of immunosuppression is linked to the dominant presence of M2 macrophages in the TME (55–57). This mechanism may also explain the low level expression of soluble, systemic co-inhibitory immune checkpoints that may result from M2 macrophage-mediated exclusion of other immunosuppressive cell types from the TME. Future studies should investigate the role of M2-like macrophages, granulocytic myeloid-derived suppressor cells (MDSCs), Tregs and other immunosuppressive cells and their relationships with soluble immune checkpoint molecules, including gene expression studies.

In conclusion, our study has revealed a seemingly novel mechanism of immune dysregulation operative in early breast cancer, which is associated with deceased plasma concentrations of both soluble co-stimulatory and co-inhibitory immune checkpoints which, is likely to emanate from a M2 macrophage-dominated, quiescent TME. Adjunctive strategies targeting M2 macrophages, either directly or indirectly, may improve outcomes in the setting of early breast cancer.

## Data Availability Statement

The raw data supporting the conclusions of this article will be made available by the authors, without undue reservation.

## Ethics Statement

The studies involving human participants were reviewed and approved by Research Ethics Committee of the Faculty of Health Sciences, University of Pretoria. The patients/participants provided their written informed consent to participate in this study.

## Author Contributions

BR, HS, SN, and RA conceptualized the study. BR, C-AB, and SN provided clinical oversight. HS, AT, NH, TS, LK, and LH were responsible for diligent processing and storage of blood/plasma specimens and for performance of laboratory analyses of plasma specimens. BR, PM, and TS advised on aspects of statistical analysis of data. All authors contributed to interpretation of data and preparation of the manuscript, and all consented to its submission.

## Funding

This study was funded by a grant awarded to BR by the Cancer Association of South Africa (CANSA).

## Conflict of Interest

Author SN is a partner at Drs Gritzman & Thatcher Inc. Laboratories.

The remaining authors declare that the research was conducted in the absence of any commercial or financial relationships that could be construed as a potential conflict of interest.

## Publisher’s Note

All claims expressed in this article are solely those of the authors and do not necessarily represent those of their affiliated organizations, or those of the publisher, the editors and the reviewers. Any product that may be evaluated in this article, or claim that may be made by its manufacturer, is not guaranteed or endorsed by the publisher.
