# Supplementary material for: Systemic Immune Dysregulation in Early Breast Cancer Is Associated With Decreased Plasma Levels of Both Soluble Co-Inhibitory and Co-Stimulatory Immune Checkpoint Molecules
Source: Front Immunol. 2022 May 23;13:823842. doi: 10.3389/fimmu.2022.823842 (PMC9168983; doi:10.3389/fimmu.2022.823842)
Supplement: Supplementary file 1 [file Table_1.docx]

**Supplementary material:**

Table S1: Correlation coefficients and *p* values showing the strength of associations of M-CSF, GDF-15, FGF-21 and CCL5 with the various immune checkpoints**.**

| **Variables** | **M-CSF** | | **GDF-15** | | **FGF-21** | | **CCL5** | |
| --- | --- | --- | --- | --- | --- | --- | --- | --- |
|  | **Spearman Correlation** | **Spearman *p-*value** | **Spearman Correlation** | **Spearman *p-*value** | **Spearman Correlation** | **Spearman *p-*value** | **Spearman Correlation** | **Spearman *p-*value** |
| GITR | -0,22 | 0,044 | -0,12 | 0,091 | 1,11 | 0,967 | 0,22 | 0,031 |
| CD27 | -0,14 | 0,538 | -0,09 | 0,323 | 0,17 | 0,411 | 0,22 | 0,035 |
| CD28 | -0,10 | 0,757 | -0,12 | 0,116 | 0,26 | 0,093 | 0,15 | 0,155 |
| CD40 | -0,11 | 0,970 | -0,05 | 0,309 | 0,2 | 0,286 | 0,18 | 0,083 |
| CD80 | -0,09 | 0,878 | -0,13 | 0,114 | 0,25 | 0,079 | 0,16 | 0,112 |
| ICOS | -0,14 | 0,698 | -0,09 | 0,194 | 0,21 | 0,170 | 0,2 | 0,051 |
| PD-1 | -0,02 | 0,401 | -0,1 | 0,141 | 0,32 | 0,008 | 0,15 | 0,151 |
| PD-L1 | -0,24 | 0,030 | -0,13 | 0,094 | 0,1 | 0,855 | 0,17 | 0,089 |
| CTLA-4 | -0,12 | 0,654 | -0,13 | 0,074 | 0,27 | 0,081 | 0,17 | 0,104 |
| TIM-3 | -0,07 | 0,328 | 0,18 | 0,055 | -0,04 | 0,897 | -0,06 | 0,55 |
| HVEM | 0,00 | 0,227 | 0,23 | 0,056 | 0,07 | 0,936 | 0,08 | 0,439 |
| TLR-2 | -0,04 | 0,606 | -0,1 | 0,135 | 0,31 | 0,012 | 0,13 | 0,217 |
| LAG-3 | -0,10 | 0,828 | -0,14 | 0,135 | 0,25 | 0,026 | 0,21 | 0,038 |
| GITRL | 0,02 | 0,297 | -0,19 | 0,220 | 0,3 | 0,003 | 0,14 | 0,179 |
| BTLA | 0,05 | 0,124 | -0,04 | 0,229 | 0,39 | 0,000 | 0,1 | 0,311 |
| CD86 | -0,06 | 0,793 | -0,11 | 0,120 | 0,29 | 0,021 | 0,17 | 0,105 |
