# Supplementary material for: Systemic Immune Dysregulation in Early Breast Cancer Is Associated With Decreased Plasma Levels of Both Soluble Co-Inhibitory and Co-Stimulatory Immune Checkpoint Molecules
Source: Front Immunol. 2022 May 23;13:823842. doi: 10.3389/fimmu.2022.823842 (PMC9168983; doi:10.3389/fimmu.2022.823842)
Supplement: Supplementary file 2 [file Table_2.docx]

**Supplementary material:**

Table S2: Correlation coefficients and *p* values showing the strength of associations of M-CSF, GDF-15, FGF-21 and CCL5 with the various cytokines and remaining chemokines.

| **Variables** | **M-CSF** | | **GDF-15** | | **FGF-21** | | **CCL5** | |
| --- | --- | --- | --- | --- | --- | --- | --- | --- |
|  | **Spearman Correlation** | **Spearman *p-*value** | **Spearman Correlation** | **Spearman *p-*value** | **Spearman Correlation** | **Spearman *p-*value** | **Spearman Correlation** | **Spearman *p-*value** |
| IL-16 | 0,35 | 0,001 | -0,10 | 0,230 | -0,32 | 0,001 | -0,04 | 0,701 |
| INF-α2 | 0,03 | 0,762 | -0,19 | 0,013 | 0,46 | 0,000 | 0,67 | 0,520 |
| IL-1RA | -0,04 | 0,711 | -0,23 | 0,100 | 0,35 | 0,000 | -0,05 | 0,661 |
| INF-y | 0,04 | 0,619 | -0,12 | 0,703 | 0,45 | 0,001 | 0,05 | 0,604 |
| IL-6 | 0,13 | 0,141 | -0,19 | 0,671 | 0,38 | 0,000 | -0,12 | 0,241 |
| IL-2 | 0,12 | 0,096 | -0,18 | 0,051 | 0,44 | 0,015 | 0,09 | 0,407 |
| IL-8 | 0,23 | 0,025 | -0,02 | 0,681 | 0,50 | 0,000 | -0,07 | 0,476 |
| IL-10 | 0,00 | 0,763 | -0,18 | 0,129 | 0,39 | 0,083 | 0,09 | 0,388 |
| IL-17A | 0,07 | 0,622 | 0,25 | 0,207 | 0,49 | 0,024 | 0,03 | 0,802 |
| IL-4 | 0,09 | 0,130 | -0,22 | 0,097 | 0,44 | 0,000 | -0,05 | 0,599 |
| CXCL5 | 0,13 | 0,024 | 0,14 | 0,058 | -0,26 | 0,042 | 0,13 | 0,208 |
| CCL23 | 0,04 | 0,800 | 0,02 | 0,625 | -0,09 | 0,754 | 0,02 | 0,815 |
| CCL26 | -0,27 | 0,044 | -0,10 | 0,448 | -0,18 | 0,419 | 0,05 | 0,629 |
| CX3CL1 | -0,08 | 0,388 | -0,07 | 0,431 | -0,07 | 0,566 | -0,03 | 0,749 |
| CXCL10 | -0,04 | 0,584 | 0,09 | 0,225 | 0,08 | 0,712 | 0,02 | 0,822 |
| CXCL9 | -0,14 | 0,328 | 0,16 | 0,335 | -0,22 | 0,883 | 0,13 | 0,222 |
